# Protocol for manufacturing the GFRP sample using VARI applied to a modified complex Arcan fixture

**DOI:** 10.1016/j.xpro.2024.103115

**Published:** 2024-06-09

**Authors:** Fefria Tanbar, Nugroho Karya Yudha, Daffa Alandro, Ariyana Dwiputra Nugraha, Eko Supriyanto, Muhammad Akhsin Muflikhun

**Affiliations:** 1 PLN Puslitbang, Jl. Duren Tiga Raya No.102, Pancoran, Kota Jakarta Selatan, Jakarta 12760, Indonesia; 2 Mechanical and Industrial Engineering Department, Gadjah Mada University, Jl. Grafika No. 2, Yogyakarta 55281, Indonesia; 3 Center of Energy Studies, Gadjah Mada University, Sekip K-1A Kampus UGM, Yogyakarta 55281, Indonesia

**Keywords:** Microscopy, Chemistry, Material sciences

## Abstract

Manufacturing techniques play an essential role in obtaining optimum mechanical properties of composites. Vacuum-assisted resin infusion (VARI) is a composite fabrication approach for optimal fiber volume fraction. Here, we present a protocol for manufacturing glass fiber-reinforced polymer (GFRP) samples by applying VARI to a modified complex Arcan fixture. We describe steps for material preparation, molding preparation, setting vacuum system, resin mixing, and degassing. We then detail procedures for vacuum infusion process and cutting composites for shear testing samples.

For complete details on the use and execution of this protocol, please refer to Alandro et al.^1^

## Highlights

• Instructions for manufacturing GFRP samples using VARI
• Steps for the preparation of the vacuum-assisted resin infusion method
• Detailed steps to prepare resin solution for VARI

## Abstract (editor highlights)

Publisher’s note: Undertaking any experimental protocol requires adherence to local institutional guidelines for laboratory safety and ethics.

## Graphical abstract

**Figure:**
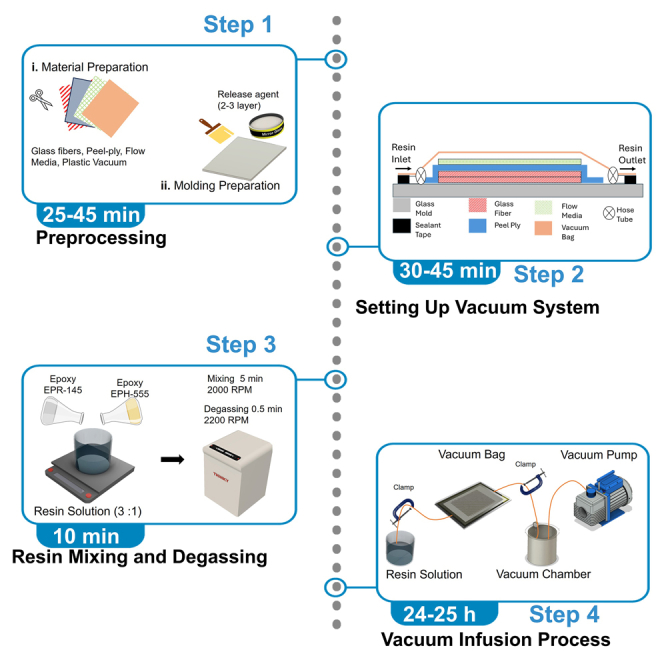

## Before you begin

This protocol describes the detailed steps for manufacturing GFRP sample using VARI applied for modified complex Arcan fixture. However, the shear tests are performed using thermoset composites with woven glass fiber reinforcement. This can also apply to thermoplastics and other types of reinforcement. Please refer to ASTM D7078^2^ for other materials that can be tested.

## Key resources table

**Table:** 

REAGENT or RESOURCE	SOURCE	IDENTIFIER
**Chemicals, peptides, and recombinant proteins**		

Epoxy resin bisphenol A-epichlorohydrin	PT Justus Kimiaraya	EPR-174
Epoxy hardener cycloaliphatic amine	PT Justus Kimiaraya	EPH-555
Release agent Miracle Gloss mold release wax	Stoner Molding Solutions, Hong Kong, China	N/A

**Other**		

Woven roving glass fiber	PT Justus Kimiaraya	200 gr/m^2^
Peel-ply	PT Justus Kimiaraya	N/A
Flow media 125–160 gr/m^2^	PT Justus Kimiaraya	N/A
Plastic vacuum bagging 75 μm thick	PT Justus Kimiaraya	N/A
Clear hose 10–12 mm	PT Justus Kimiaraya	N/A
Spiral warp/tube 10–12 mm	PT Justus Kimiaraya	N/A
T-Connector 10–12 mm	PT Justus Kimiaraya	N/A
Sealant tape/Tacky tape	PT Justus Kimiaraya	N/A
Vacuum pump	Zhejiang Value Co., Ltd.	VP80-1HP
Universal testing machine	Carson Tech. Co., Ltd.	CRN-50
Microscope Dino-Lite Edge 1.3MP	Dunwell Tech, Inc.	AF4915
Planetary centrifugal mixer	Thinky Corporation	ARE-310

## Step-by-step method details

### Material preparations for VARI

**Timing: 10 min**

The important thing about this material preparation is the gap between the glass fibers, peel-ply, flow media, and vacuum plastic. The gap between the other materials is 5–10 cm. In this protocol, the size used illustrates the fabrication of a 25 × 25 cm composite. For applications in composite fabrication with different sizes, the material gap can be used as a ref. 3.

**CRITICAL:** Working with glass fiber can irritate the skin and eyes. Use gloves, coats, and goggles. Keep glass fiber in a closed container at room temperature.

1. Cutting and arranging glass fiber.
  a. Measure the glass fibers that will be cut to 25 × 25 cm. To obtain a good cut result, use paper tape on the glass fibers that will be cut. See Figure 1A as an example.
    ***Note:*** When measuring glass fibers, use a tolerance of approximately 1–2 cm because the composites will be cut 1 cm from the end.

**Figure 1:**
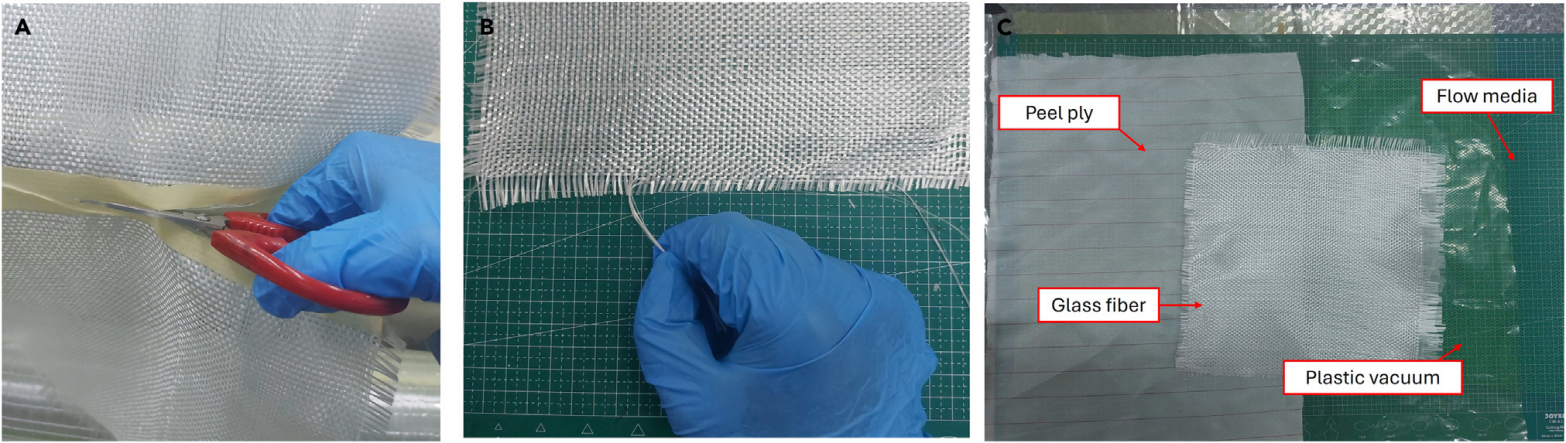
Preparation of materials
  b. Cut the glass fibers using scissors. Remove the remnants of glass fiber pieces because they can potentially trigger leakage during the VARI process (Figure 1B).
2. Preparation of peel-ply, flow media, and plastic vacuum (Figure 1C).
  a. Cut peel-ply with a size of 30 × 35 cm.
  b. Cut flow media with a size of 20 × 25 cm.
  c. Cut plastic vacuum with a size of 40 × 40 cm, the size of the plastic is made loose so that it is not pulled during the vacuum process.
3. Preparation of the hose and spiral tube.
  a. Cut 50 cm of hose used as resin inlet and outlet.
  b. Cut 50 cm long spiral tube and cut it into 4 pieces.
  c. Attach the spiral tube to the T-Connector.

**Figure 2:**
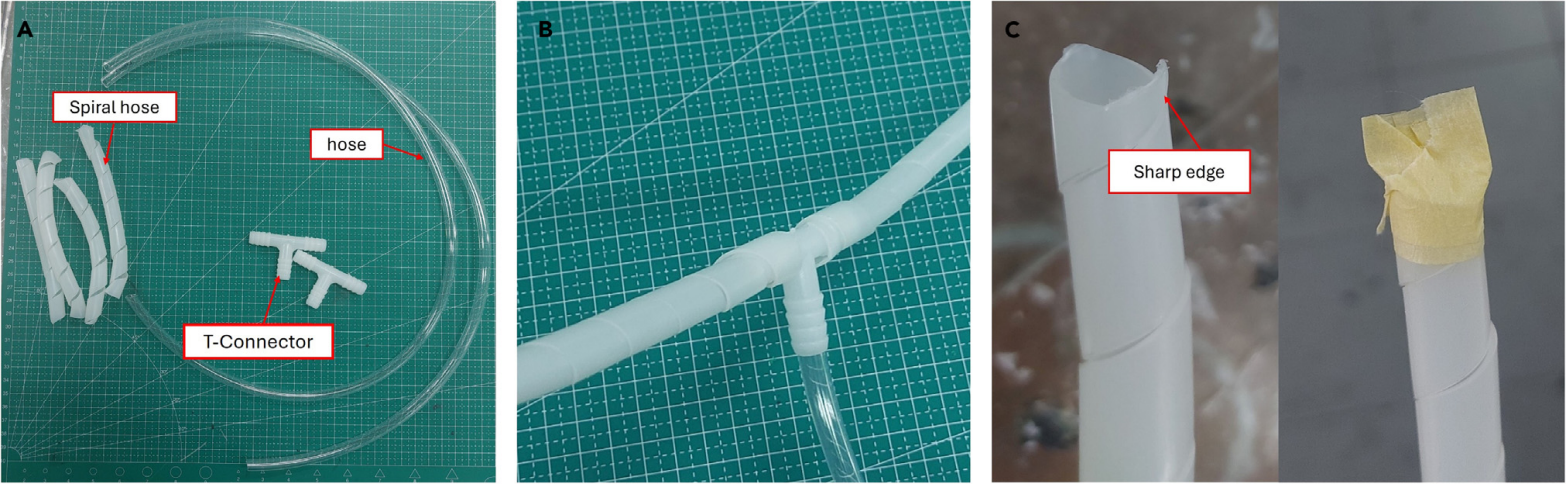
Resin inlet and outlet preparation

**CRITICAL:** The spiral tube has a sharp edge that can damage the plastic vacuum. Use paper tape to cover the edge. See Figure 2C as an example.

### Molding preparation

**Timing: 15–30 min**

The mold used is a flat plate made of glass. The mold can use other materials, such as aluminum, silicone, and ceramic, but the point is that the material has a smooth, non-stick surface. If a new mold is used, for the application of the release agent apply 5 coats of release agent. For specific applications and the usage of different release agents please follow the application instructions on the product.

4. Mold cleaning and preparation.
  a. Clean the mold surface with a putty knife.
  b. Apply release agent on the mold surface. Apply 2 or 3 coats of release agent with a clean towel using circular motions. Allow 3–5 min between applications/coats. See Figure 3 as an example.

**Figure 3:**
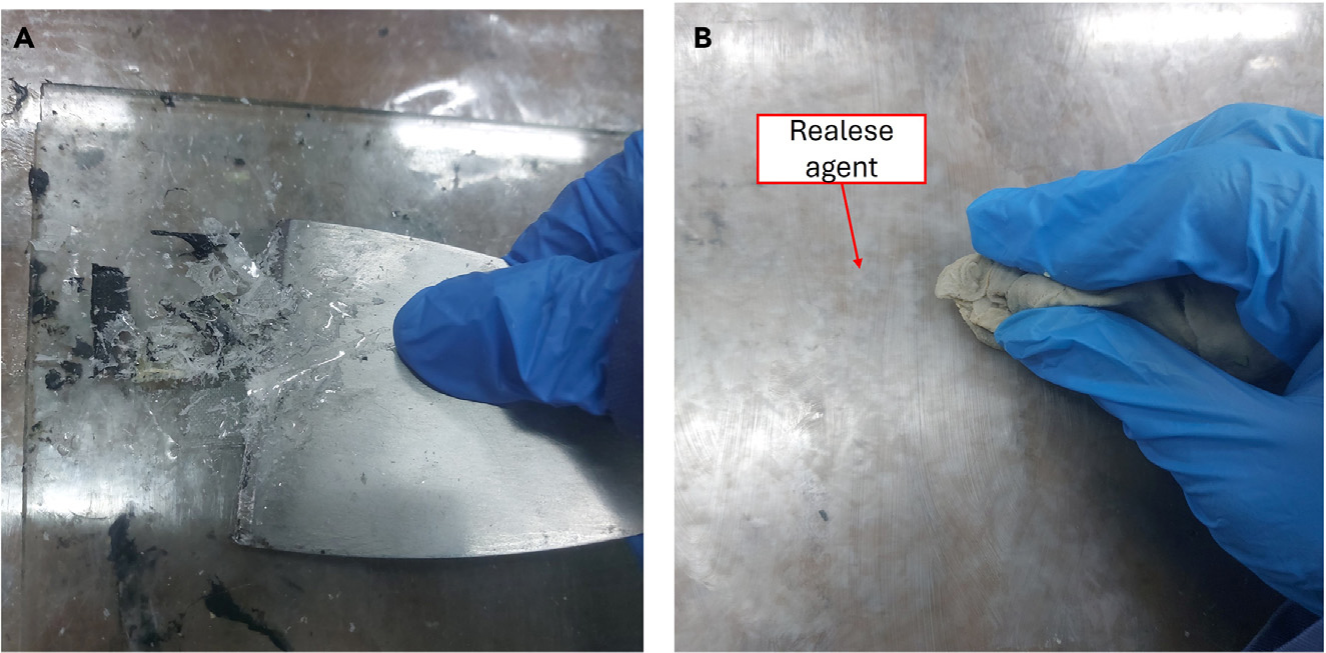
Mold cleaning and preparation
5. Placement of vacuum-assisted resin infusion materials.
  a. Apply 2 sheets of glass fiber with orientation of 0/90 degrees. troubleshooting 1.
  b. Apply the peel-ply above the glass fiber, then the flow media on top of the peel-ply.
  c. Set the position of the material as shown in Figure 4A.

**Figure 4:**
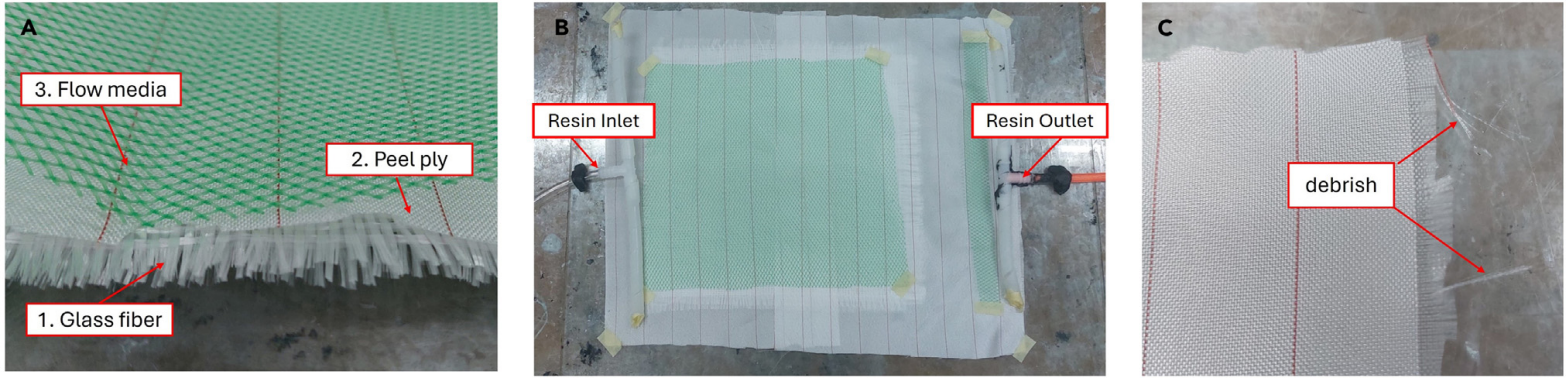
Positioning material and hose (A) Material laying sequence. (B) Position of inlet and outlet. (C) Example of remnants derived from peel-ply.

***Note:*** Remove the remnants of fibers and other remnants from the mold, this helps prevent leakage. See Figure 4C as an example.

6. Install vacuum ports and tubes.
  a. Set the position of the assembly of the spiral tube as an inlet and outlet for resin distribution media.
  b. Use seal tape to attach the assembly to the mold.

### Setting up the vacuum system

**Timing: 30–45 min**

When preparing the vacuum bag, create a loose space between the material and the vacuum plastic so that there is enough space when the vacuum plastic is pulled during the vacuum process. If possible, use double seal tape in crucial parts such as the connection between the hose and vacuum plastic, folds on the vacuum plastic and areas that are felt necessary.^4^

**CRITICAL:** This section involves dangerous pressurized equipment. Ensure the seals on the vacuum pump and resin trap are correctly installed.

7. Installing vacuum bag.
  a. Apply seal tape to the vacuum plastic.
  b. Install the vacuum plastic into the mold starting from the hose section, then give a little distance so the plastic is not pulled during the vacuum process. See Figure 5A as an example.

**Figure 5:**
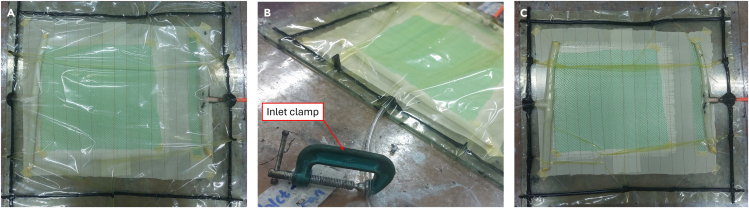
Process of checking leakage of the vacuum bag
8. Install the port and the hose to the vacuum chamber. Ensure the hose installation is not rigid so as not to remove the attached sealant tape potentially.
9. Examine the leakage of the vacuum bag.
  a. Clamp inlet hose.
  b. Turn on the vacuum pump until the pressure in the vacuum bag reaches −1 MPa.
  c. After 10 min, check for leaks in the vacuum bag and the hose installation in the vacuum chamber. If there is no leakage, turn the valve lever on the vacuum chamber and turn off the vacuum pump. troubleshooting 2.

### Resin mixing and degassing

**Timing: 10 min**

This section describes the procedure to generate the resin solution used in the VARI method. In preparing resin and hardener solution for vacuum-assisted resin infusion, the ratio used is 3:1.^5^ The mixing and degassing process uses a Thinky machine. Other alternatives can be used, such as manually stirring and degassing using a vacuum chamber.

10. Prepare the scale.
11. Prepare Thinky containers for resin and hardener.
12. Pour 120 g of resin and 40 g of hardener into the Thinky container.
13. Mix and defoam the resin solution using Thinky machine. troubleshooting 3.
  a. Put the container in the Thinky machine.
  b. Set the Thinky lever according to the gross weight of the container. As shown in Figure 6C.

**Figure 6:**
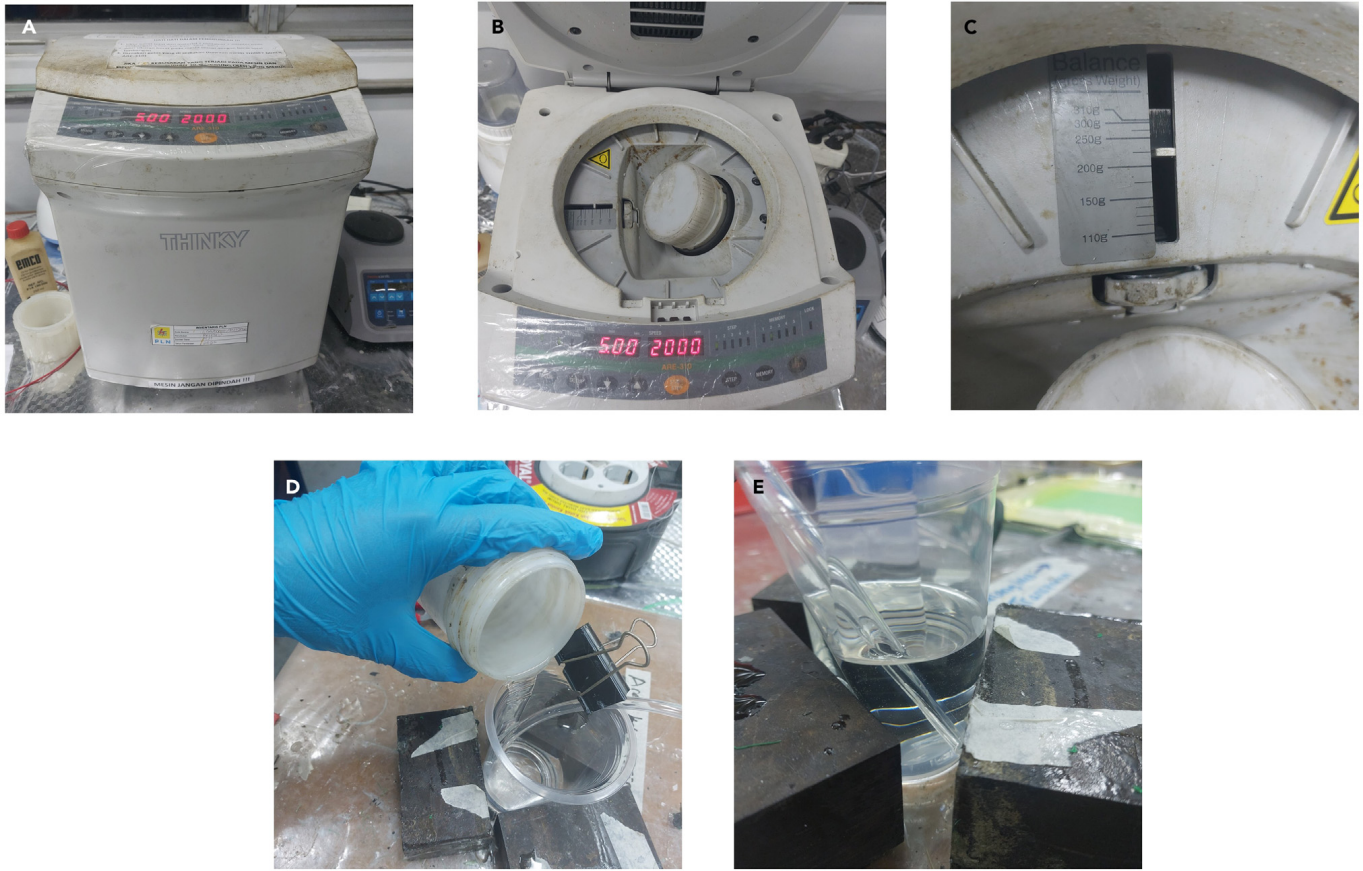
Resin mixing and degassing process
  c. Set the step mode for 5 min at 2000 RPM and the defoaming mode for 30 s at 2200 RPM.
14. When finished, remove it from the Thinky machine and pour it slowly into the provided container.

### Vacuum infusion process

**Timing: 24–25 h**

This section describes the procedure for conducting resin infusion into the molding. Using a ratio of 3:1 and a mixing time of 5 min, the working time for the resin mixture to reach the peak temperature is 30 min. For complete details on different resin ratios and mixing times, please refer to Fadlurrahman et al.^6^

15. Ensure the inlet hose is firmly attached to the resin container.
16. Turn on the vacuum pump and open the clamp in the resin inlet.
17. The resin will flow from the resin inlet to the resin outlet during the vacuum process. troubleshooting 4.
18. Monitor the resin infusion process. troubleshooting 5.
  a. During the resin infusion process, monitor the resin flow until the resin wets the entire material. See Figure 7 as an example.

**Figure 7:**
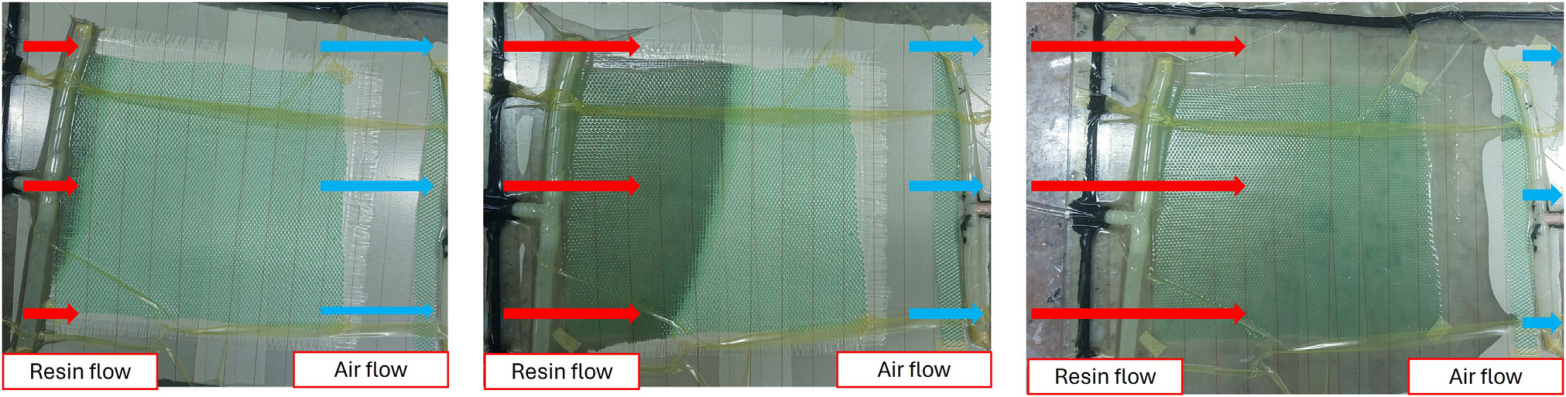
Resin infusion process
  b. Also check if any voids can be removed by prolonging the vacuum process.^7^
19. After the vacuum infusion process is complete, clamp the resin inlet to the resin outlet, rotate the lever on the vacuum chamber, and turn off the vacuum pump. Wait for curing for 24 h at a 20°C–25°C temperature. See Figure 8 as an example.

**Figure 8:**
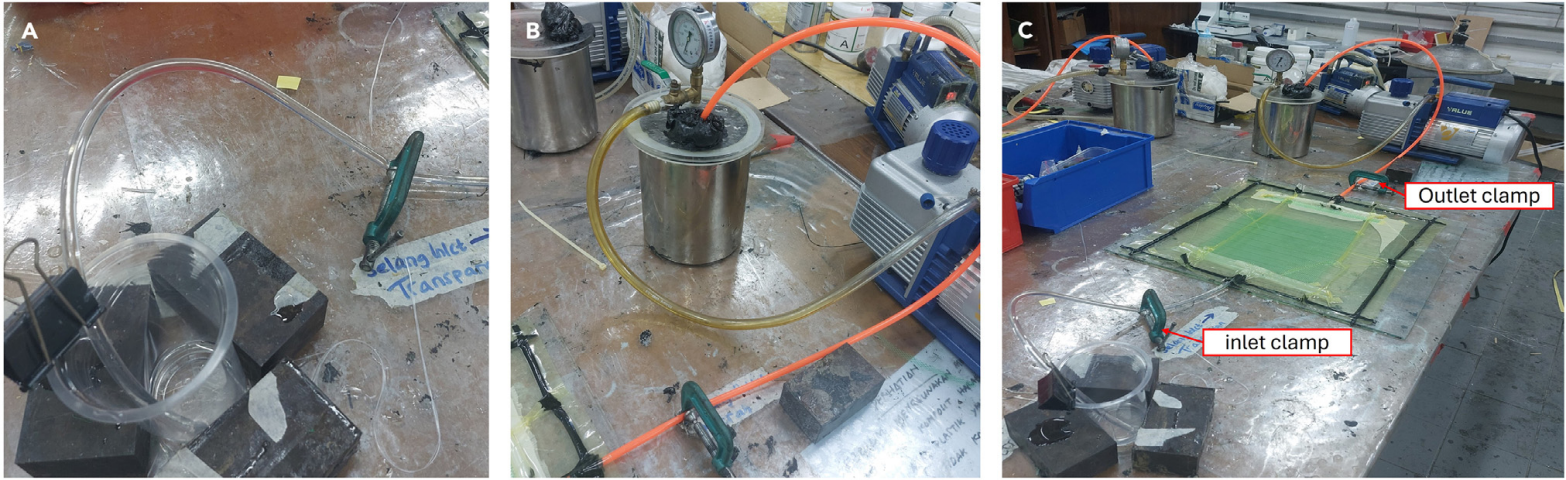
Completed resin infusion process

### Post-infusion finishing

**Timing: 3–45 min**

In this section, the composite produced by the VARI method is separated from the mold and finished for optimal results.

20. Pull the vacuum plastic from the mold and cut out the resin inlet and outlet.
21. Remove the composite from the mold by pulling the peel-ply, then separate the composite from the peel-ply. See Figure 9 as an example.

**Figure 9:**
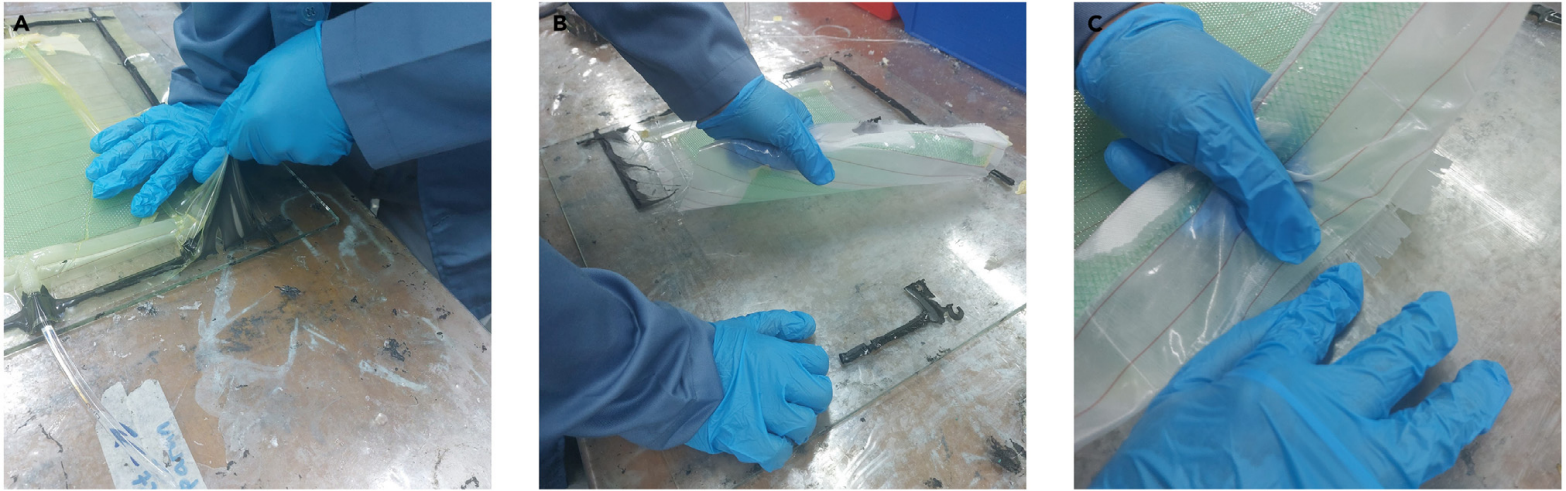
Removing the composite from the molding and vacuum bag
22. Trimming and Sanding.
  a. Draw the specimen pattern on the composite using a marker pen and then cut the composite according to the pattern. See Figure 10 as an example. troubleshooting 6.

**Figure 10:**
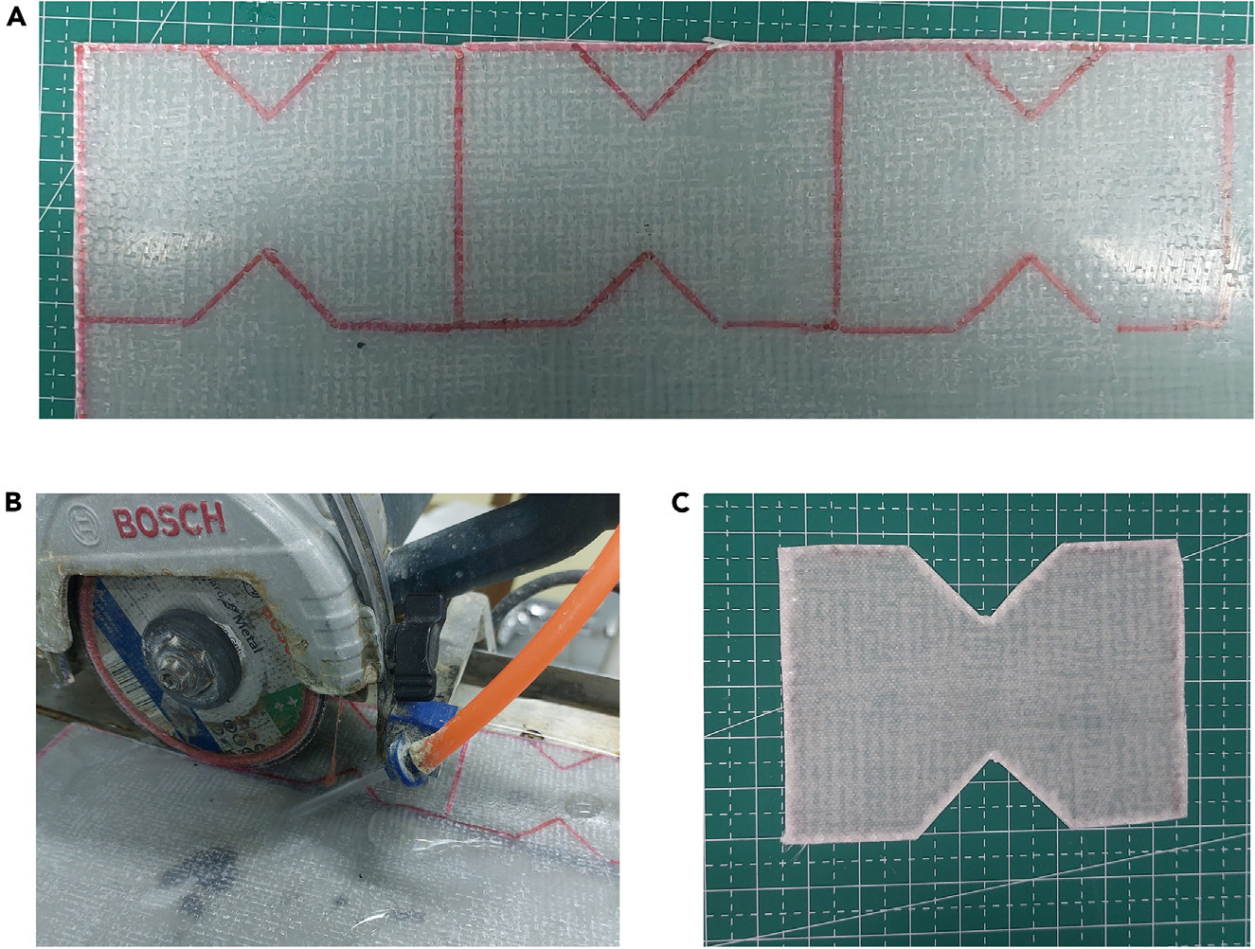
Trimming and sanding composite
  b. Use sandpaper to make the cut smoother.

### Mechanical testing

**Timing: 45 min**

Mechanical evaluation of the fabricated composites was performed using a universal testing machine. This evaluation included shear tests performed with an Arcan fixture,^8^^,^^9^^,^^10^ following ASTM D7078 - D7078M. An in-depth description of the calculation methods used in the tests, statistical results, and equipment used in the tests are explained in the following sections: alat, load displacemet, failure.

**Figure 11:**
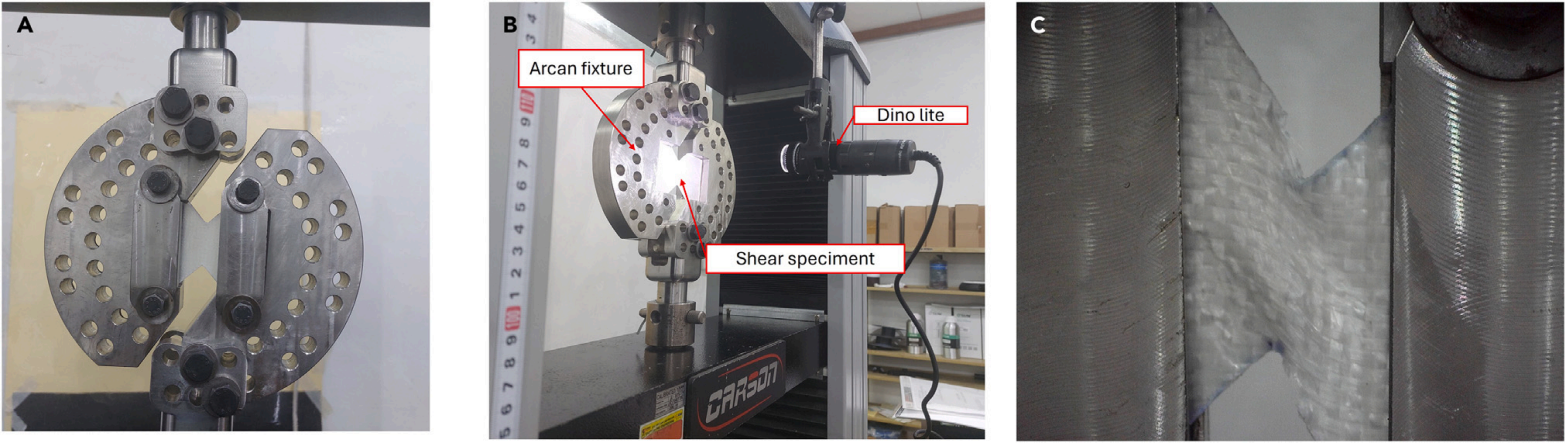
Arcan fixture shear test equipment setup and result (A) Arcan fixture shear configuration. (B) Arcan fixture experimental setup. (C) Sample condition during testing. Shear test experimental result of GFRP Composite.

***Note:*** Shear stress and ultimate shear strength are calculated by:

23. Perform shear testing according to ASTM D7078 - D7078M.
  a. Perform the test with at least five specimens per test condition.
  b. Use a loading rate of 2 mm/min.
24. Use the Dino-Lite AF4915 microscope with 20–200× magnification to observe the shear phenomenon during the test. See Figure 11 as an example.

τ=P/2A

τ = shear strength, (MPa);

P = Applied load, (N);

A = cross-sectional area, (mm^2^);

***Note:*** shear strain and ultimate shear strain are calculated by:

$$\gamma =\epsilon_{x}-\;\epsilon_{y}$$

γ = shear strain;

$\epsilon_{x}$ = Longitudinal normal strain;

$\epsilon_{y}$ = Lateral normal strain;

***Note:*** shear modulus is calculated by:

$$G^{chord}=\Delta \tau /\Delta \gamma$$

$G^{chord}$ = shear cord modulus of elasticity (GPa);

Δτ = difference in applied shear stress between the two strain points;

Δγ = difference in applied shear stress between the two engineering strain points;

## Expected outcomes

Using this protocol, you will be able to produce uniformly infused GFRP panels with low void content, and an appropriate fiber volume fraction. These qualities are crucial since they directly impact the composite’s mechanical properties and overall performance. For mechanical testing, at least five specimens per test condition should result in data points that enable calculating these mechanical properties, ensuring robust statistical analysis. Additionally, observing the shear phenomenon via a Dinolite microscope during testing will provide visual insight into the failure mechanisms of the GFRP composites.

## Limitations

When fabricating composites using vacuum-assisted resin infusion, there are several limitations, the most important of which is the size of composite fabrication. If the size is too large, it can affect the resin infusion time. Resin has a limited working time before it starts to gel. If the infusion time exceeds the working time, the resin may not thoroughly wet the fiber reinforcement. In addition, not all types of resins can be applied to VARI. If the viscosity of the resin is too high, the resin may not flow adequately through the material, leading to incomplete infusion or dry spots.

## Troubleshooting

### Problem 1

Fiber misalignment during laying up.

### Potential solution

Fiber orientation is essential in composite fabrication. There are often errors or changes in fiber orientation/misalignment during preparation. Before laying the glass fiber on the mold, make sure the orientation of the glass fiber direction is correct, then apply paper tape to prevent fiber misalignment.

### Problem 2

Leakage in the Vacuum System.

### Potential solution

Leaks can be detected using a leak detector or observing the pressure gauge for 10–15 min. If there is a leak, there will be a pressure drop, either drastically or slowly, on the pressure gauge on the resin trap. Recheck all connections, seal tape installation, and ensure the vacuum bag is leak-free. Seal tape installation can also be observed by inverting the glass mold.

### Problem 3

Workability time of resin solution.

### Potential solution

The mixing and degassing process length will affect the workability time of the resin during the vacuum infusion process. Degassing can reduce voids in the mixed solution. However, a longer degassing duration will decrease the workability time of the resin, which should be considered when producing large composite products. This can be overcome by manually mixing the resin solution before mixing and degassing using a Thinky machine.

### Problem 4

Low fiber volume fraction.

### Potential solution

It is critical to manufacture composites that have high fiber volume fraction. However, if the initial pressure of VARI is not reached, the fiber volume fraction will decrease due to the resin trapped in the laminate. Ensure the initial pressure reaches – 1 MPa before the resin flows through the laminates.

### Problem 5

Insufficient resin during the resin infusion process.

### Potential solution

Monitoring the resin flow during the infusion process is critical to ensure complete material wetting and identify resin insufficiency in the resin container. Adjust the duration of the vacuum process if a resin insufficiency is detected by clamping the resin inlet, reducing the flow of the vacuum pump, and then adding resin solution to the resin container.

### Problem 6

burnt results from the cutting process using grinders.

### Potential solution

Burning often occurs when cutting composites using grinders, which can potentially affect the properties of the fabricated composites. Use running water as a coolant when cutting composites. For detailed parts, use a mini grinder, and finish by sanding the cut area using sandpaper with 600 and 800 grit.

## Resource availability

### Lead contact

Further information and requests for resources and reagents should be directed to and will be fulfilled by the lead contact, Muhammad A. Muflikhun (akhsin.muflikhun@ugm.ac.id).

### Technical contact

Technical questions on executing this protocol should be directed to and will be answered by the technical contact, Nugroho Karya Yudha (nugrohokaryayudha@mail.ugm.ac.id).

### Materials availability

This study did not generate new unique materials.

### Data and code availability

• All data reported in this paper will be shared by the lead contact upon request.
• This paper does not report the original code.
• Any additional information required to reanalyze the data reported in this paper is available from the lead contact upon request, Muhammad A. Muflikhun (akhsin.muflikhun@ugm.ac.id).
